# Collateral Effects of Insecticide-Treated Nets on Human and Environmental Safety in an Epidemiological Model for Malaria with Human Risk Perception

**DOI:** 10.3390/ijerph192316327

**Published:** 2022-12-06

**Authors:** Juan Pablo Gutiérrez-Jara, Katia Vogt-Geisse, Maritza Cabrera

**Affiliations:** 1 Centro de Investigación de Estudios Avanzados del Maule (CIEAM), Vicerrectoría de Investigación y Postgrado, Universidad Católica del Maule, Talca 3480112, Chile; 2 Facultad de Ingeniería y Ciencias, Universidad Adolfo Ibáñez, Santiago 7941169, Chile

**Keywords:** mathematical epidemiology, malaria, insecticide-treated nets, insecticide exposure, risk perception, ecosystem damage, mosquito net fishing, impulsive differential equations

## Abstract

Malaria remains a major health problem in many parts of the world, including Sub-Saharan Africa. Insecticide-treated nets, in combination with other control measures, have been effective in reducing malaria incidence over the past two decades. Nevertheless, there are concerns about improper handling and misuse of nets, producing possible health effects from intoxication and collateral environmental damage. The latter is caused, for instance, from artisanal fishing. We formulate a model of impulsive differential equations to describe the interplay between malaria dynamics, human intoxication, and ecosystem damage; affected by human awareness to these risks and levels of net usage. Our results show that an increase in mosquito net coverage reduces malaria prevalence and increases human intoxications. In addition, a high net coverage significantly reduces the risk perception to disease, naturally increases the awareness for intoxications from net handling, and scarcely increases the risk perception to collateral damage from net fishing. According to our model, campaigns aiming at reducing disease prevalence or intoxications are much more successful than those creating awareness to ecosystem damage. Furthermore, we can observe from our results that introducing closed fishing periods reduces environmental damage more significantly than strategies directed towards increasing the risk perception for net fishing.

## 1. Introduction

Malaria is a vector-borne disease caused by intracellular parasites of the genus *Plasmodium*. Five species are known to infect humans: *P. falciparum*; *P. vivax*; *P. ovale*; *P. malariae*; and *P. knowlesi* [1,2]. The disease is mainly transmitted into the human blood by the bites of female *Anopheles* mosquitoes, who feed for survival and the production of eggs; thereby, the infective sporozoites from their salivary glands are passed to the human circulation system and multiply inside its red blood cells [1,2]. Other mechanisms of malaria transmission are via blood transfusion and congenitally [3,4]. Children under 5 years of age are most vulnerable to malaria, as is also the case for other vector-borne diseases [1,5]. Additionally, malaria remains a major cause of perinatal mortality, maternal anemia, and low birth weight [3,6].

Malaria is a major health problem, mainly affecting tropical and subtropical counties, causing 241 million cases worldwide in 2020 and 627,000 deaths [1,7]. This disease is endemic in 85 countries and the WHO African region accounted for 95% of the global cases in 2020 [8]. Despite a significant effort made by the WHO and other international agencies in the fight against malaria, its control still remains a public health challenge [8].

The use of insecticide-treated nets (ITNs), in conjunction with chemical spraying and larval management, has proven to be an effective preventive mechanism used against malaria [2,3,9,10]. In 2020, 229 million ITNs were delivered to malaria-endemic countries, mainly located in Sub-Saharan Africa, with a reported figure of 65% of the households using at least one ITN and 43% of the population sleeping under ITNs [7]. It has been reported that ITNs reduce child mortality by 17% compared to no nets usage, saving 5.6 lives each year for every 1000 children worldwide, as well as a 44% reduction in the incidence of severe malaria episodes in the ITNs group, among others [11,12]. Preventive and treatment measures to reduce the burden of the disease also include the use of antimalarial drugs, vaccination of children in moderate- to high-transmission regions, and vector-control strategies [1].

Undoubtedly, the distribution and use of ITNs have made a huge impact on the control and prevention of malaria worldwide; nevertheless, there exist increasing concerns over the misuse of bed nets for artisanal fisheries, driven, among other reasons, by the necessity to ensure food security to local communities [13,14,15]. The fine mesh size of ITNs allows for a substantial removal of aquatic species located in coastal habitats, e.g., in seagrass meadows. These harbor a diverse and abundant population of juveniles that might play an important ecological role [14,16,17]. The fishing of these species—easily accessible to coastal communities in emerging economies—may contribute to the already-present damage due to overfishing and the loss of ecosystem functioning. Hence, it may produce critical ecological implications threatening the sustainability of fish stocks in different parts of the world [14,16,18]. Most affected by ITN fishing are juvenile fish, seahorses, marine shrimp species, common silver biddy, milk fish, silver cyprinid, and Lake Malawi sardine, among others [16]. However, the information regarding the effects of ITNs for fishing on inhabiting or co-inhabiting species is still scarce [14], and more studies are needed in that field, especially since ITN fishing has been observed globally. In fact, it has been detected across all equatorial continents, including locations in Sub-Saharan Africa, several countries and regions in Asia and Oceania, and some in Latin America, affecting marine and freshwater habitats [16].

Studies have indicated that ITNs are safe to use if properly handled, and potential health risks are likely below acceptable threshold values [19,20,21]. However, it is important to be aware of possible effects that ITNs may have on people’s health since pesticides, by nature, are responsible for adverse effects such as psychomotor damage, malformations in fetuses, and cancer, among others [22,23,24,25,26]. A study stated that the insecticide *permethrin*, used to treat mosquito nets, was considered a neuropoison, because of its adverse effects on the nervous system, also causing itching and burning effects on exposure skin, in addition to direct inhalation [27]. Another study also revealed that there is some potential for adverse health effects due to mishandling of ITNs, especially for infants and toddlers, however, under conservative exposure scenarios [21]. Hence, there have been concerns regarding possible health effects due to pyrethroids exposure from ITNs use, but potential toxicity of frequent or long-term exposure to ITNs in malaria-endemic countries has little been studied on site [28,29,30]. In addition, there may be potential of indirect poisoning of humans when ITNs are used for fishing, since ITNs are not intended to be submerged in water. In particular, the leaking of harmful concentrations of insecticide to aquatic ecosystems—which can be extremely toxic to fish [20]—raises the question of whether bioaccumulation of pyrethroids in fish for human consumption may have a detrimental effect on human health [14].

Compartmental mathematical models for vector-borne diseases are vastly found in the literature [31,32,33], and are mainly based on and inspired by the Ross–McDonald malaria model [34]. In particular, there is a significant number of articles associated with mathematical modeling of malaria dynamics [35,36,37,38,39,40]. The behavior of the disease in humans is generally expressed through an SEIR-type model, while the vector follows SI-type dynamics [36,37,40]. As mentioned before, one of the leading mitigation measures over time has been the use of ITNs, which has had a significant impact in the reduction of malaria cases, in combination with other mitigation strategies [1,7,12,14,41]. This result has been supported and analyzed by mathematical models [35,42,43,44,45,46].

Malaria dynamics and the implementation of mitigation measures occur at different timescales, which may be difficult to implement through classical models of ordinary differential equations. Impulsive differential equations, through their hybrid systems, are capable of modeling these dynamics with greater precision [47,48,49]. There are some studies that have used these models to describe malaria dynamics, for instance, to express impulsive releases of sterile mosquitoes [49] or to describe a pulse strategy to control mosquitoes through insecticide spraying [48]. Other articles have included pulse effects regarding intoxications and their effects on people’s health [25,50]. The novelty of our work is to model, through this type of equations, the dynamics of malaria, pulse human intoxication, and pulse environmental effects due to the use and misuse of ITNs. Additionally, these dynamics are affected by different levels of risk perceptions, which determine the awareness of humans towards malaria disease, intoxication from ITNs, and environmental damage.

The structure of this paper is as follows. In Section 2, we present and explain the mathematical model of impulsive differential equations. In Section 3, we generate numerical simulations to observe the trajectories corresponding to malaria infection, human ITN intoxication, and ecosystem damage due to misuse of ITNs as fishing gear, and how these are impacted through risk perception and ITN coverage levels. Finally, in Section 4, we discuss our results and propose future directions.

## 2. Materials and Methods

**Figure 1:**
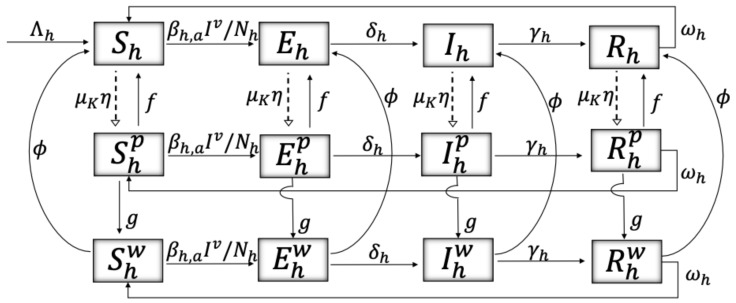
$I^{v}=I_{v}+I_{v}^{r}$. Schematics showing the flow between malaria disease states: Susceptible ($S_{h}^{x}$), exposed ($E_{h}^{x}$), infectious ($I_{h}^{x}$), and recovered ($R_{h}^{x}$); the flow between intoxication levels due to insecticide exposure, where the superscript x∈{{},p,w} represents poison states in humans: no toxicity, acute intoxication, and toxicity with possible health effects (chronic intoxication), respectively. The dashed lines represent discrete moments in time when individuals are exposed to the toxicity, and the solid lines represent continuous rates. A description of the variables and parameters used can be found in Table 1 and Table 2, respectively.

We present a mathematical model of impulsive differential equations that include malaria dynamics in humans and mosquitoes, two types of intoxication in humans produced by ITNs exposure, and two states representing the environment and its damage due to misuse of ITNs. Figure 1 depicts malaria dynamics in humans, dividing the total human population ($N_{h}$) into susceptible ($S_{h}$), exposed ($E_{h}$), infectious ($I_{h}$), and recovered ($R_{h}$). The superscript of each of these states (see Figure 1) represents the level of toxicity in humans: no superscript representing no toxicity, *p* representing acute intoxication, and *w* representing possible health effects after prolonged exposure (chronic intoxication). Malaria is transmitted to susceptible humans by infected mosquitoes, $I_{v}^{r}$ and $I_{v}$, insecticide-resistant and non-resistant mosquitoes, respectively, in continuous time (solid lines) according to a force of transmission $\beta_{h,a}\left(t\right)I^{v}\left(t\right)/N_{h}\left(t\right)$, with $I^{v}=I_{v}+I_{v}^{r}$, where the subscript *h* represents transmission from mosquitoes to humans and *a*, with a∈{1,2,3,4} indicating four transmission levels that may differ annually due to seasonal mosquito presence and may be location-dependent. The transmission rate is defined as

(1) $$\beta_{h,a}\left(t\right)=\beta_{h,a}^{*}\left(1-\eta \right)K_{d}^{*}/K_{d}\left(t\right),$$

where $\beta_{h,a}^{*}$ is the natural transmission rate, η is the ITN coverage, and $K_{d}\left(t\right)$ is a variable representing risk perception of humans to malaria disease, such that an increase in risk perception from a natural risk perception to malaria, $K_{d}^{*}$, results in a reduction of the transmission rate. The dynamics of this risk variable are determined by the following differential equation, as in [25,51,52,53]:

(2) $$\dot{K_{d}}\left(t\right)=-\lambda_{1}^{d}\left(K_{d}-K_{d}^{*}\right)+\lambda_{2}^{d}\left(I_{h}+I_{h}^{p}+I_{h}^{w}\right)/N_{h},$$

where $\lambda_{1}^{d}$ represents resistance to change and $\lambda_{2}^{d}$ represents the velocity of reaction to the presence of disease. We observe that individuals increase their risk perception to malaria when there is an increase in the number of infectious individuals. Humans transition from exposed to infectious at a rate $\delta_{h}$, recover at a rate $\gamma_{h}$, and lose immunity at a rate $\omega_{h}$. Insecticide intoxication occurs in discrete time (segmented lines), considering that exposure to the toxic substance happens during handling or through direct contact with ITNs. When these events happen, a proportion $\mu_{K}\eta :=\mu \left(K_{p}^{*}/K_{p}\left(t\right)\right)\eta$ of individuals become acutely intoxicated (superscript *p*), where $K_{p}\left(t\right)$ is a dynamic variable representing peoples’ risk perception regarding health effects from exposure to insecticides against mosquitoes, in bed-nets. The higher the risk perception is, the less intoxication occurs. The dynamics of this risk perception variable are given by the following differential equation:

(3) $$\dot{K_{p}}\left(t\right)=-\lambda_{1}^{p}\left(K_{p}-K_{p}^{*}\right)+\lambda_{2}^{p}\sum_{X}\left(X_{h}^{p}+X_{h}^{w}\right)/N_{h},\;X\in \left\{S,E,I,R\right\},$$

which is similar to Equation (2) and where $K_{p}^{*}$ represents the natural risk perception to health damage due to insecticide exposure in the population. We observe that individuals increase their risk perception when there is presence of intoxicated individuals. Acutely intoxicated individuals can present, over time, chronic intoxication with possible health effects, and hence can transition from the $X_{h}^{p}$ states to the $X_{h}^{w}$ states at a rate *g*, with X∈{S,E,I,R}. Detoxification from both intoxication states occurs at rates *f* and ϕ, respectively.

All individuals are born disease-free and intoxication-free at a rate $\Lambda_{h}$. Mortality occurs at a rate $d_{h}$ in all states, and there is malaria-disease-induced mortality for infectious individuals occurring at a rate ${\hat{d}}_{h}$. Note that we did not include the mortality rates in Figure 1 in order to preserve the visual clarity of the schematics.

**Figure 2:**
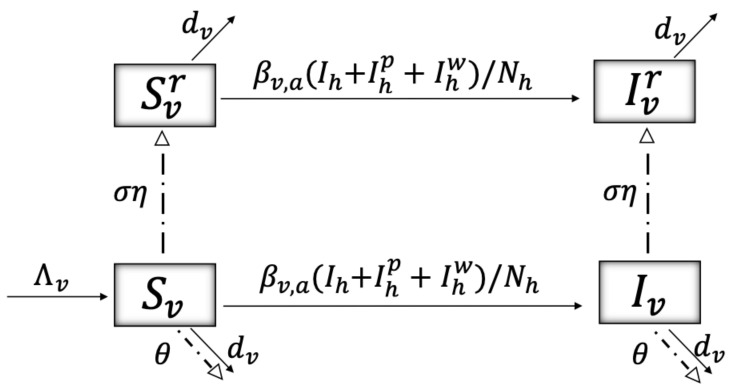
Schematics showing the flow between malaria disease states for mosquitoes: susceptible, $S_{v}^{r}$ and $S_{v}$, and infectious, $I_{v}^{r}$ and $I_{v}$, representing insecticide-resistant and non-resistant mosquitoes, respectively. The dashed-dotted lines represent discrete moments in time when mosquitoes are exposed to the insecticide, and the solid lines represent continuous rates. A description of the variables and parameters shown can be found in Table 1 and Table 2, respectively.

Figure 2 shows the malaria dynamics of mosquitoes, dividing their population into susceptible, $S_{v}^{r}$ and $S_{v}$, and infectious, $I_{v}^{r}$ and $I_{v}$, representing insecticide-resistant and non-resistant mosquitoes, respectively. Mosquitoes become infected by infectious humans according to a force of transmission $\beta_{v,a}\left(t\right)\left(I_{h}\left(t\right)+I_{h}^{p}\left(t\right)+I_{h}^{w}\left(t\right)\right)/N_{h}\left(t\right)$, where the transmission rate is defined as $\beta_{v,a}\left(t\right)=\beta_{v,a}^{*}\left(1-\eta \right)K_{d}^{*}/K_{d}\left(t\right)$, with $\beta_{v,a}^{*}$ being the natural transmission rate from humans to mosquitoes, $K_{d}\left(t\right)$ the risk perception to malaria of humans as in Equation (2), and a∈{1,2,3,4} representing four transmission rates that may be seasonally and location-dependent, as mentioned before. Mosquitoes may die at a rate $d_{v}$. We omitted incubation time, loss of immunity, and disease-induced death for mosquitoes due to their short lifespan. One of the novelties of our model is that it includes a pulse, modeling the exposure of the vectors to the ITNs (dash-dotted line), assuming two possibilities: (i) mosquitoes can acquire some level of resistance to the toxic substance, transitioning from the $Y_{v}$ states to the $Y_{v}^{r}$ states, Y∈{S,I}, such that a fraction ση does so; (ii) the insecticide satisfies its purpose, increasing the mortality rate of mosquitoes by a proportion θ. Mosquitoes are recruited into the susceptible class $S_{v}$ without being insecticide-resistant, at a rate $\Lambda_{v}$.

**Figure 3:**
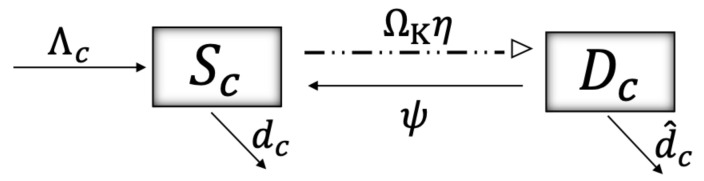
The schematic shows the flow between the states of an aquatic ecosystem: susceptible to damage from ITNs fishing ($S_{c}$) and damaged from ITNs fishing ($D_{c}$). The transition from susceptible-to-damage to damaged occurs through a pulse at moments triggered by ITNs fishing (represented by a dashed-dotted-dotted line), and solid lines represent continuous rates. A description of the variables and parameters used can be found in Table 1 and Table 2, respectively.

Repurposing and misuse of ITNs, for instance, for fishing, is of increasing concern since it can harm aquatic ecosystems due to the capture of species that play an important ecological role, which may lead to overfishing and the loss of ecosystem functioning. Here, we call “collateral damage” or “environmental damage” the detrimental effect of ITN fishing on the aquatic ecosystem, leading to an increase in the mortality rate of its species. We model that damage considering two states for the aquatic ecosystem: susceptible to damage ($S_{c}$) and damaged ($D_{c}$), such that the damaged ecosystem has a higher mortality rate (${\hat{d}}_{c}>d_{c}$). Figure 3 shows the schematics of the collateral damage flow. The moments (pulses) when a proportion $\Omega_{K}\eta$ of susceptible species in the ecosystem are damaged correspond to instants when ITNs are being used for fishing (dashed-dotted-dotted line). $\Omega_{K}:=\Omega \left(K_{c}^{*}/K_{c}\left(t\right)\right)$, where Ω is a constant and $K_{c}\left(t\right)$ is a dynamic variable that represents the risk perception of humans to the collateral damage produced by ITN fishing, such that the higher the risk perception, the less damage that occurs. The dynamics of this risk variable are given by the following differential equation:

(4) $$\dot{K_{c}}\left(t\right)=-\lambda_{1}^{c}\left(K_{c}-K_{c}^{*}\right)+\lambda_{2}^{c}\left({\hat{d}}_{c}D_{c}\right)/N_{c},$$

where $K_{c}^{*}$ represents the natural risk perception to collateral damage of individuals and $N_{c}:=S_{c}+D_{c}$. From Equation (4) we can observe that individuals increase their risk perception to collateral damage as a reaction to the mortality of the damaged ecosystem. Damaged species may recover at a rate ψ, and we assume a constant nondamaged recruitment into the ecosystem at a rate $\Lambda_{c}$.

The model described by the schematics in Figure 1, Figure 2 and Figure 3 is a model of coupled impulsive differential equations given in system (5). $t_{i}$ and $t_{j}$ correspond to instants at which humans and mosquitoes are exposed to the toxicity emitted by the mosquito net, respectively. $t_{n}$ corresponds to instants at which humans create damage to aquatic ecosystems due to the misuse of ITNs. At those time points, the malaria transmission rates $\beta \in \{\beta_{h,a},\beta_{v,a}\}$ increase their value by a factor α, since using ITNs for fishing entails not using them for malaria protection. A description of the variables and parameters used can be found in Table 1 and Table 2, respectively.

(5) $$\left\{ \begin{array}{l} \begin{array}{ccc} \left.\begin{array}{ccc} \dot{S_{h}}\left(t\right) & =\Lambda_{h}-\beta_{h,a}\left(t\right)S_{h}\left(I_{v}+I_{v}^{r}\right)/N_{h}+fS_{h}^{p}+\omega_{h}R_{h}+\phi S_{h}^{w}-d_{h}S_{h}\\ \dot{E_{h}}\left(t\right) & =\beta_{h,a}\left(t\right)\left(I_{v}+I_{v}^{r}\right)/N_{h}+fE_{h}^{p}+\phi E_{h}^{w}-\left(\delta_{h}+d_{h}\right)E_{h}\\ \dot{I_{h}}\left(t\right) & =\delta_{h}E_{h}+fI_{h}^{p}+\phi I_{h}^{w}-\left(\gamma_{h}+d_{h}+{\hat{d}}_{h}\right)I_{h}\\ \dot{R_{h}}\left(t\right) & =\gamma_{h}I_{h}+fR_{h}^{p}+\phi R_{h}^{w}-\left(\omega_{h}+d_{h}\right)R_{h}\\ \; & \;\\ \dot{S_{h}^{p}}\left(t\right) & =-\beta_{h,a}\left(t\right)S_{h}^{p}\left(I_{v}+I_{v}^{r}\right)/N_{h}+\omega_{h}R_{h}^{p}-\left(f+g+d_{h}\right)S_{h}^{p}\\ \dot{E_{h}^{p}}\left(t\right) & =\beta_{h,a}\left(t\right)S_{h}^{p}\left(I_{v}+I_{v}^{r}\right)/N_{h}-\left(\delta_{h}+f+g+d_{h}\right)E_{h}^{p}\\ \dot{I_{h}^{p}}\left(t\right) & =\delta_{h}E_{h}^{p}-\left(\gamma_{h}+f+g+d_{h}+{\hat{d}}_{h}\right)I_{h}^{p}\\ \dot{R_{h}^{p}}\left(t\right) & =\gamma_{h}I_{h}^{p}-\left(f+g+\omega_{h}+d_{h}\right)R_{h}^{p}\\ \; & \;\\ \dot{S_{h}^{w}}\left(t\right) & =-\beta_{h,a}\left(t\right)S_{h}^{w}\left(I_{v}+I_{v}^{r}\right)/N_{h}+gS_{h}^{p}+\omega_{h}R_{h}^{w}-\left(\phi +d_{h}\right)S_{h}^{w}\\ \dot{E_{h}^{w}}\left(t\right) & =\beta_{h,a}\left(t\right)S_{h}^{w}\left(I_{v}+I_{v}^{r}\right)/N_{h}+gE_{h}^{p}-\left(\delta_{h}+\phi +d_{h}\right)E_{h}^{w}\\ \dot{I_{h}^{w}}\left(t\right) & =\delta_{h}E_{h}^{w}+gI_{h}^{p}-\left(\gamma_{h}+\phi +d_{h}+{\hat{d}}_{h}\right)I_{h}^{w}\\ \dot{R_{h}^{w}}\left(t\right) & =\gamma_{h}I_{h}^{w}+gR_{h}^{p}-\left(\phi +\omega_{h}+d_{h}\right)R_{h}^{w}\\ \; & \; & \\ \dot{S_{v}}\left(t\right) & =\Lambda_{v}-\beta_{v,a}\left(t\right)S_{v}\left(I_{h}+I_{h}^{p}+I_{h}^{w}\right)/N_{h}-d_{v}S_{v}\\ \dot{S_{v}^{r}}\left(t\right) & =-\beta_{v,a}\left(t\right)S_{v}^{r}\left(I_{h}+I_{h}^{p}+I_{h}^{w}\right)/N_{h}-d_{v}S_{v}^{r}\\ \dot{I_{v}}\left(t\right) & =\beta_{v,a}\left(t\right)S_{v}\left(I_{h}+I_{h}^{p}+I_{h}^{w}\right)/N_{h}-d_{v}I_{v}\\ \dot{I_{v}^{r}}\left(t\right) & =\beta_{v,a}\left(t\right)S_{v}^{r}\left(I_{h}+I_{h}^{p}+I_{h}^{w}\right)/N_{h}-d_{v}I_{v}^{r}\\ \; & \; & \end{array}\right.\\ \;\dot{S_{c}}\left(t\right)=\Lambda_{c}+\psi D_{c}-d_{c}S_{c}\\ \dot{D_{c}}\left(t\right)=-\psi D_{c}-{\hat{d}}_{c}D_{c}\\ \; & \; & \\ \dot{K_{p}}\left(t\right)=-\lambda_{1}^{p}\left(K_{p}-K_{p}^{*}\right)+\lambda_{2}^{p}\sum_{X}\left(X_{h}^{p}+X_{h}^{w}\right)/N_{h}\\ \dot{K_{d}}\left(t\right)=-\lambda_{1}^{d}\left(K_{d}-K_{d}^{*}\right)+\lambda_{2}^{d}\left(I_{h}+I_{h}^{p}+I_{h}^{w}\right)/N_{h}\\ \dot{K_{c}}\left(t\right)=-\lambda_{1}^{c}\left(K_{c}-K_{c}^{*}\right)+\lambda_{2}^{c}\left({\hat{d}}_{c}D_{c}\right)/N_{c} \end{array}\}\;t\notin \left\{t_{i},t_{j},t_{n}\right\}\\ \begin{array}{cc} X_{h}\left(t^{+}\right) & =\left[1-\mu \left(K_{p}^{*}/K_{p}\right)\eta \right]X_{h}\\ X_{h}^{p}\left(t^{+}\right) & =X_{h}^{p}+\mu \left(K_{p}^{*}/K_{p}\right)\eta X_{h} \end{array}\}t=t_{i},\;X\in \{S,E,I,R\}\\ \begin{array}{cc} Y_{v}\left(t^{+}\right) & =\left(1-\sigma \eta \right)Y_{v}\\ Y_{v}^{r}\left(t^{+}\right) & =Y_{v}^{r}+\sigma \eta Y_{v}^{m}\\ d_{v}\left(t^{+}\right) & =\left(1+\theta \right)d_{v} \end{array}\}t=t_{j},\;Y\in \{S,I\}\\ \begin{array}{cc} S_{c}\left(t^{+}\right) & =\left[1-\Omega \left(K_{c}^{*}/K_{c}\right)\eta \right]S_{c}\\ D_{c}\left(t^{+}\right) & =D_{c}+\Omega \left(K_{c}^{*}/K_{c}\right)\eta S_{c}\\ \beta (t^{+}) & =(1+\alpha )\beta \; \end{array}\}t=t_{n},\;\beta \in \{\beta_{h,a},\beta_{v,a}\} \end{array}\right.$$

## 3. Simulations

**Figure 4:**
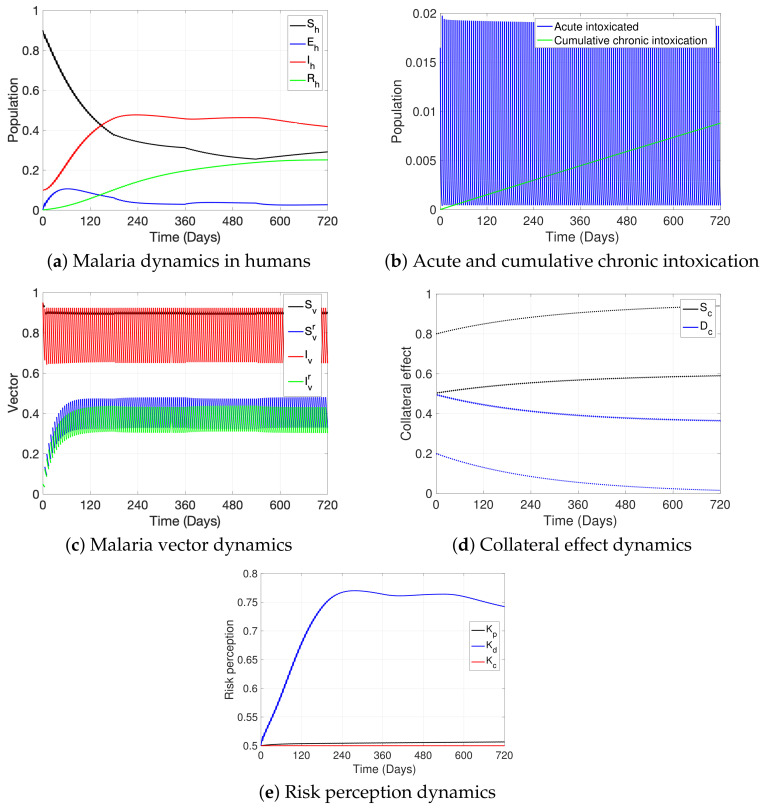
The figure depicts the basic dynamics of the model: (**a**) depicts the behavior over time of the epidemiological classes of malaria in humans; (**b**) shows the point prevalence of acute intoxication cases (blue) and the cumulative chronic intoxication cases (green) over time; (**c**) shows the behavior over time of the epidemiological classes of malaria in mosquitoes; (**d**) depicts the dynamics of the collateral damage of the ecosystem; and (**e**) shows the risk perception dynamics over time to toxicity of ITNs ($K_{p}$), malaria disease ($K_{d}$), and to collateral damage ($K_{c}$). The parameter values used are the following. For humans: $\beta_{h,1}=\beta_{h,2}=0.022*\left(1-\eta \right)$, $\beta_{h,3}=\beta_{h,4}=0.015*\left(1-\eta \right)$, $\delta_{h}=1/15$, $\gamma_{h}=1/200$, $\omega_{h}=8.3*10^{-3}$, μ=0.033, f=0.75, g=0.00289, ϕ=0, $\Lambda_{h}=d_{h}=3.3*10^{-5}$, ${\hat{d}}_{h}=9.01*10^{-5}$, $\lambda_{1}^{p}=0.05$, $\lambda_{1}^{d}=0.07$, $\lambda_{1}^{c}=0.09$, $\lambda_{2}^{p}=0.02$, $\lambda_{2}^{d}=0.04$, $\lambda_{2}^{c}=0.01$, $K_{p}^{*}=K_{d}^{*}=K_{c}^{*}=0.5$. For mosquitoes: $\beta_{v,1}=\beta_{v,2}=0.022*\left(1-\eta \right)$, $\beta_{v,3}=\beta_{v,4}=0.015*\left(1-\eta \right)$, σ=0.2, $\Lambda_{v}=d_{v}=1/15$, θ=0.2. For collateral: Ω=0.75, $\Lambda_{c}=d_{c}=8.4*10^{-4}$, ${\hat{d}}_{c}=dc+21*10^{-4}$, $\psi =4.2*10^{-3}$, α=0.2. Initial condition: $\left(0.9,0.0,0.1,0.0,0.0,0.0,0.0,0.0,0.0,0.0,0.0,0.0,0.5,0.0,0.5,0.0,0.8,0.2,K_{p}^{*},K_{d}^{*},K_{c}^{*}\right)$, η=0.5, $\Delta t_{i,j,n}=5$.

**Figure 5:**
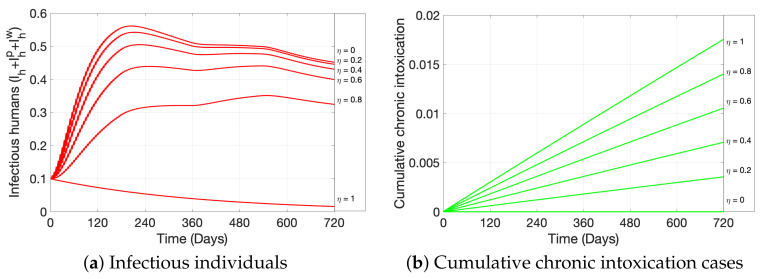
The figure shows the effect of ITN coverage η=0,0.2,0.4,0.6,0.8,1 on (**a**) point prevalence of malaria-infected humans and (**b**) cumulative chronic intoxication cases after a prolonged exposure to ITNs. All other parameter values used are as in Figure 4.

**Figure 6:**
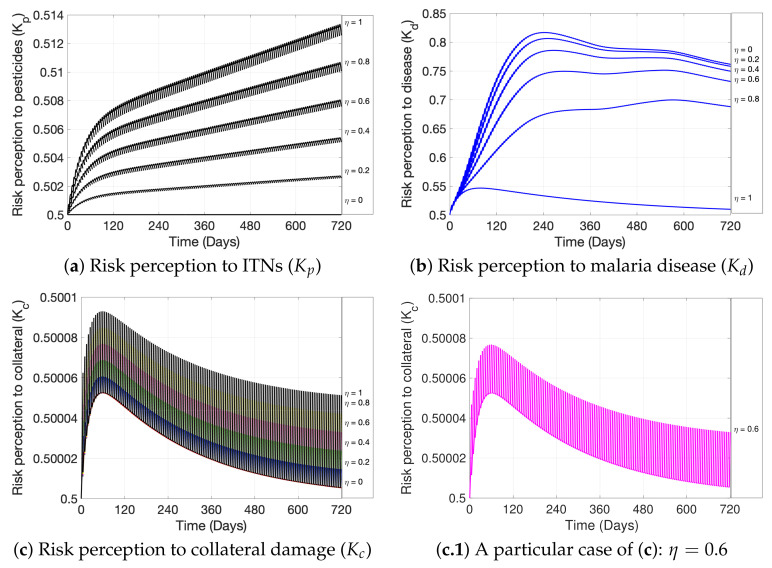
The figure shows, for the values of ITNs coverage η=0,0.2,0.4,0.6,0.8,1, its effect on the dynamics of (**a**) risk perception to toxicity of ITNs, $K_{p}$; (**b**) risk perception to malaria disease, $K_{d}$; (**c**) risk perception to collateral damage due to misuse of ITNs, $K_{c}$; (**c.1**) is a particular case of (**c**), picturing risk perception to collateral for η=0.6. All other parameter values used are as in Figure 4.

**Figure 7:**
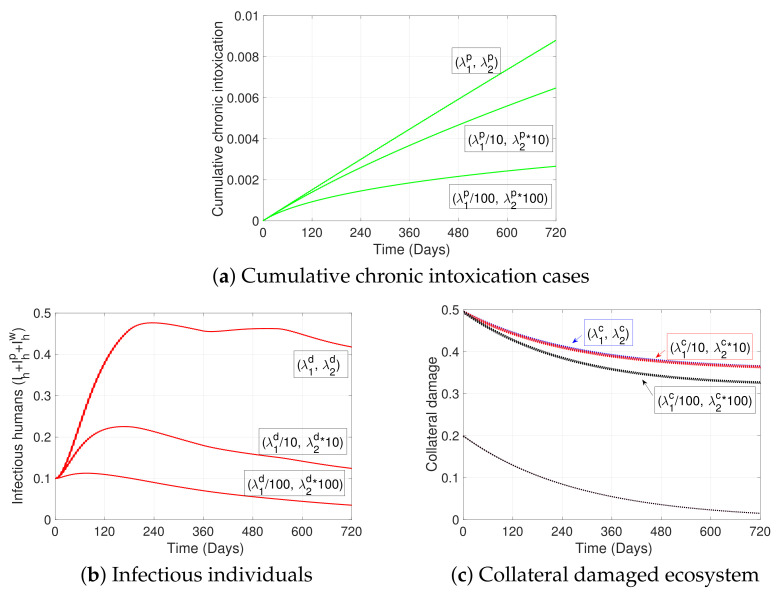
The figure shows the effect of risk perception on (**a**) cumulative chronic intoxication after long-term ITNs exposure, (**b**) malaria disease, and on (**c**) collateral damage ($D_{c}$) of the ecosystem due to misuse of ITNs as fishing gear. The parameters $\lambda_{1}^{k}$ and $\lambda_{2}^{k}$, with k=p,d,c, were reduced and increased by a factor of 10 and 100, respectively. All other parameter values used are as in Figure 4.

**Figure 8:**
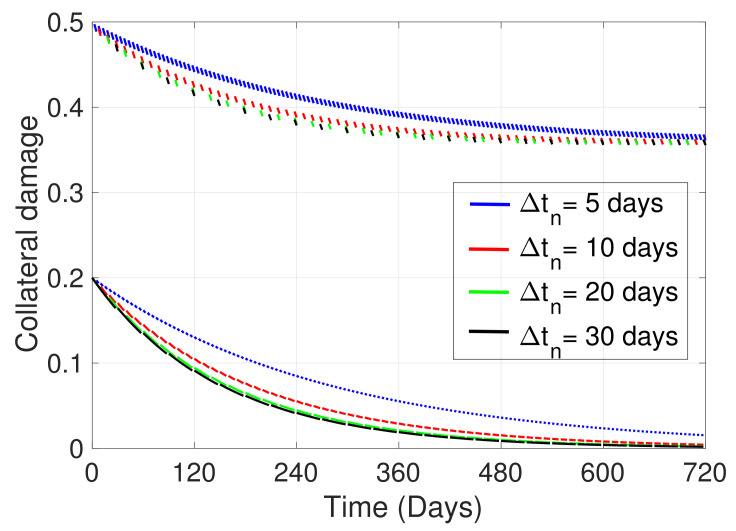
The figure shows the collateral damage of the ecosystem due to misuse of ITNs as fishing gear for different no-fishing timeframes $\Delta t_{n}$ days. The blue, red, green, and black lines correspond to average no-fishing timeframes of 5, 10, 20, and 30 days, respectively. All other parameter values used are as in Figure 4.

In this section, we first present, in Figure 4, the general dynamics of the model; second, in Figure 5, we analyze the effect of ITNs coverage (η) on disease dynamics and on chronic intoxication, and in Figure 6 how different levels of ITN coverage affect the risk perception of humans to disease, intoxication, and collateral damage; third, in Figure 7, we show how increasing risk perceptions affect disease dynamics, intoxication, and collateral damage over time; finally, in Figure 8, we study the effect of fishing restrictions on collateral damage to aquatic ecosystems. The general dynamics of the different compartments of the model are presented in Figure 4.

Figure 4a shows the behavior over time of the epidemiological classes of malaria in humans, reaching an endemicity level. Figure 4b depicts the proportion of overall acute intoxication cases in humans at any particular time (blue) and the cumulative number of cases with chronic intoxication that may lead to possible health effects after exposure to ITNs (green). We assume that acute intoxications occur in discrete time ($\Delta t_{i}=5$ days); this situation is observed in the figure when looking at the respective jumps, while, on the contrary, the cumulative chronic intoxications are represented by a continuous curve.

Figure 4c shows the general dynamics of the epidemiological classes of malaria for mosquitoes. We observe jumps, which explicitly represent the exposure of mosquitoes to the toxicity of ITNs in discrete time ($\Delta t_{j}=5$ days). It should also be noted that over time, the number of vectors that acquire resistance to the toxin increases (green and blue curves). Figure 4d shows the possible collateral damage produced by ITN fishing over time. In particular, the damaged ecosystem, $D_{c}$, is represented by the two blue dotted curves, picturing two damage levels that alternate at different instants, simultaneously altering the susceptible ecosystem level, $S_{c}$, represented by the black curves. More specifically, the instants at which ITN fishing happens are visualized through the jumps in the diagram ($\Delta t_{n}=5$ days, pulses happening between the pulses at $t_{i}$ and $t_{j}$), such that at every moment when fishing occurs, the level of damage, $D_{c}$, of the ecosystem increases (higher pulses in the blue curve), while $S_{c}$ decreases (lower pulses in the black curve).

Figure 4e shows the curves of risk perception towards damage to health due to the toxic exposure to ITNs ($K_{p}$, black), to disease ($K_{d}$, blue), and to collateral damage due to the misuse of ITNs for fishing ($K_{c}$, red), with the risk perception to disease being the highest among the three, followed by the risk perception to health effects due to ITN exposure.

Figure 5 depicts different levels of ITN coverage (η) in malaria-endemic regions, affecting disease dynamics and intoxication dynamics. More specifically, Figure 5a shows the proportion of infectious humans for levels of coverage between 0% (η=0) and 100% (η=1), which decreases with increasing coverage; while Figure 5b depicts the cumulative intoxication cases over time for the same ITN coverage levels, which increase with increasing coverage.

Figure 6 shows how ITN coverage levels between 0% (η=0) and 100% (η=1) affect risk perception of humans to (a) toxicity from ITN use, (b) malaria disease, and (c) collateral damage due to misuse of ITNs. Specifically, Figure 6a shows a directly proportional behavior between ITN coverage and level of risk perception to ITN exposure, $K_{p}$, i.e., the risk perception increases with increasing coverage; the same can be observed in Figure 6c for the risk perception to collateral damage, $K_{c}$. On the contrary, the risk perception towards malaria disease, $K_{d}$, behaves indirectly proportional to ITN coverage, as pictured in Figure 6b. We observe that η=0 represents the case of no ITNs availability in the population, and as soon as η>0, the pulses in the model are activated, which indirectly has a pulse effect on the risk perceptions.

Figure 7 shows the effect of different risk perception levels on (a) the cumulative cases of chronic intoxications after exposure to ITNs, (b) the proportion of malaria-infected individuals, and on (c) the collateral damage ($D_{c}$) on the ecosystem from misuse of ITNs as fishing gear. The risk perception was changed, making a decimal variation to the resistance to change parameter ($\lambda_{1}$) and the parameter ($\lambda_{2}$) that represents the reaction speed of people against the threat in each risk case. It is observed that after decreasing the resistance to change ($\lambda_{1}^{p}$) and increasing the reaction speed ($\lambda_{2}^{p}$), there is a significant decrease in the cumulative cases of chronic intoxications after exposure to ITNs (see Figure 7a). A significant decrease can also be observed regarding the number of malaria-infectious humans when applying the same decimal variation in the behavioral parameters of the risk perception towards malaria ($\lambda_{1}^{d}$ and $\lambda_{2}^{d}$) (see Figure 7b). Finally, when applying the variation to the λs of the risk perception to collateral damage ($\lambda_{1}^{c}$ and $\lambda_{2}^{c}$), a less pronounced reduction of collateral damage can be observed (see Figure 7c). Specifically, in Figure 7c, it can be seen that after the decimal variation in the behavioral parameters regarding ecosystem damage, the respective baselines—which is when ITNs are not used for fishing—coincide for the $\lambda_{1}^{c}$ and $\lambda_{2}^{c}$ values used (lower dotted line), representing low levels of ecosystem damage. On the contrary, when fishing with ITNs, pulses are activated (jumps from the baseline, every $\Delta t_{n}=5$ days between $t_{i}$ and $t_{j}$), and high levels of damage are observed (upper dotted lines). However, there is no significant difference in the collateral damage caused by ITN fishing under different levels of risk perception, since the difference between the upper lines is small.

Finally, Figure 8 shows the effect of imposing fishing restrictions, in particular limiting ITN fishing. We observe that increasing the duration of closed fishing periods, of length $\Delta t_{n}$, produces, on average, lower levels of damage to the aquatic ecosystem (average between lower lines and upper lines for each color), and also a spacing out of the dashes of the higher curves, representing time intervals with large collateral damage in each case. Hence, the longer the closed fishing periods, the lower the average collateral damage level and the shorter the timeframes producing large damage to the ecosystem.

## 4. Discussion

Malaria is a vector-borne disease transmitted by *Anopheles* mosquitoes and caused by parasites of the genus *Plasmodium*. It represents a major health problem, with Sub-Saharan Africa being most seriously affected. Insecticide-treated nets (ITNs) were mass-delivered to endemic countries and have without doubt—in combination with insecticide residual spraying—proven instrumental in the successful reduction of malaria incidence over the past two decades [1]. ITNs, if properly handled, are, in general, safe to use, and potential health risks are likely below an acceptable threshold [19,20,21]. Nevertheless, due to the poisonous nature of insecticides and the lack of more studies, there have been concerns about the potential for acute and chronic intoxication from ITN exposure [27,28,29,30]. Additionally, there have been recent discussions about the damaging effect of ITN fishing on fish stocks and aquatic biodiversity, driven often by the necessity of communities to ensure food security [13,14,15,16,17,18]. We present a compartmental model of impulsive differential equations that studies the dynamics of malaria disease, acute and chronic intoxications in humans due to pulse ITN exposure, and collateral effects on ecosystems due to pulse ITN fishing. Each of these dynamics depends on a dynamic variable representing, respectively, risk perception to disease, toxicity, and collateral damage.

Our results show that, as expected, malaria prevalence decreases with increasing ITN coverage. In particular, an 80% coverage or more produces a significant decrease in malaria prevalence (see Figure 5a) and significantly lowers the risk perception to disease (see Figure 6b). On the contrary, the number of cumulative chronic intoxication cases increases with net coverage, almost doubling the cases with twice the coverage (see Figure 5b), representing a possible health risk from ITN exposure in populations with high ITN coverage and incorrect handling practices of nets. Nevertheless, we observe from Figure 6a that, as coverage of ITNs increases and as time passes, the risk perception towards intoxication naturally increases, slightly fluctuating due to the indirect effect of pulse exposure to toxicity. This result describes a population that, with time, naturally becomes more aware of the importance of proper handling of nets and the dangers of toxicity, while the level of awareness is proportional to the level of ITN coverage. The importance of the level of risk perception towards intoxication can, in fact, be seen in Figure 7a; to exemplify, we can observe from the figure that under a scenario of a net coverage of 50%, a higher risk perception towards intoxication produces a significant decrease in cumulative chronic intoxications in the population. The latter highlights the importance of educational campaigns that teach responsible handling of ITNs (reducing the urge to return to a low risk perception; see $\lambda_{1}^{p}$ in Equation (3)) and informs about potential health effects of intoxications (increasing the reaction velocity to intoxication case count; see $\lambda_{2}^{p}$ in Equation (3)), when ITNs are mass-distributed.

We can also observe from Figure 7b, and at a net coverage of 50%, that disease awareness campaigns—which aim to reduce the urge of individuals to return to a low level of awareness and to increase the reaction velocity to malaria point prevalence (see $\lambda_{1}^{d}$ and $\lambda_{2}^{d}$ in Equation (2))—significantly decrease the point prevalence of malaria. If those campaigns are kept, a sudden increase in malaria cases could be controlled more efficiently, even with a lower ITN coverage. For disease-preparedness purposes, it may be beneficial to maintain disease awareness campaigns, even at a high ITN coverage level of more than 80%, since under such coverage, the risk perception of individuals to disease is naturally reduced significantly (see Figure 6b). The latter is related to the fact that if net coverage increases, the number of those infected with malaria decreases, so the human population “relaxes” (see Figure 5b and Equation (2)).

Regarding the collateral effect produced by ITN fishing on the aquatic ecosystem, we can observe that the collateral damage decreases with time (see Figure 4d). This is due to the decrease in the overall number of species, since the mortality rate is higher in the damaged ecosystem, resulting in fewer species to harm. The increase in the susceptible ecosystem (not damaged) is associated with the fact that the collateral damage is not permanent, and only occurs when ITNs are used for fishing. The risk perception towards collateral damage slightly increases with increasing ITN coverage (see Figure 6c), but to a much lower extent than the risk perceptions towards disease or intoxications (compared with Figure 6a,b). In fact, due to the large jumps in the risk perception to collateral damage observed in Figure 6c), overlapping between cases of coverage, and due to the small scale, there is almost no variability in the average risk perception to collateral damage for different ITN coverage levels. Hence, a mass distribution of ITNs does not have a significant impact on the risk perception of individuals to ecosystem harm, despite its dynamics being dependent on the mortality of the damaged ecosystem (see Equation (4)), which becomes damaged through pulse ITN fishing.

On the other hand, from Figure 7c we can see that a change in risk perception—by, for instance, increasing the awareness of fish mortality through ITN fishing—does not reduce the collateral damage significantly. In particular, we can even observe that the baseline for collateral damage (time intervals where ITN fishing is not performed) coincides for different levels of risk perception to damage. Hence, ecosystem damage awareness campaigns might not have a large effect on the reduction of collateral damage. This is contrary to what we have observed for malaria awareness and intoxication awareness campaigns and their respective effects on disease and intoxication case reduction. However, introducing management measures, such as implementing closed periods for fishing, does further reduce overall collateral damage and may help to ensure a sustainable use of fisheries. We observe especially that the longer the closed periods are, the longer the damage is kept at a low level (see longer dashes on lower lines in Figure 8). We point out that by applying such fishing restrictions, the overall collateral damage would be reduced, even with a high ITN coverage and, hence, a high availability of ITNs that makes the occurrence of ITN fishing more likely. However, the implementation of closed periods has proven to be challenging, since it leads to decreased fish trading activities, declining opportunities for small-scale fisheries, etc., affecting coastal livelihoods [69], and hence need to be analyzed considering socioeconomic impacts. Finally, we observe that strictly overseeing that fishing nets comply with the allowed mesh size—not affected through fishing the reproductive cycle of fish—would have an equivalent effect to increasing the length of closed periods, and hence may reduce ecosystem damage.

The main limitations of our work are the following: (i) there is little information available in the literature regarding the damage to aquatic ecosystems caused by ITN fishing, which is needed to model ecosystem damage more accurately; (ii) more information and studies are required to better understand the potentiality of health risks associated with ITN intoxication levels; (iii) additionally, more studies are needed to include in the model the loss of toxicity and efficacy of mosquito nets after being used for fishing; and (iv) the risk perception variables of our model do not consider explicitly psychological emotional factors, which may affect its dynamics. However, the generality of our model is capable of providing a framework and important qualitative results that in a novel way consider collateral effects of ITNs on human and environmental safety, considering human risk perception; hence, it is a contribution to the literature and may help to generate guidelines for decision-making in public and environmental health.

This work and our model leave a range of possibilities for future interdisciplinary work, such as (i) studying the impact of ITN fishing on the health of species inhabiting an aquatic ecosystem, in particular by analyzing the potential leaking of insecticides [14]; (ii) helping to analyze specific fishing management measures and their associated socioeconomic impacts and biological outcomes [69]; (iii) studying potential health effects in humans from the consumption of poisonous fish due to ITN fishing, and how this may affect human risk perception to intoxication; (iv) including a spatial variable in the model, etc. Finally, it is also worth mentioning that including people’s risk perception or behavior within disease modeling is a dynamic aspect to consider in future work. This behavioral aspect alters the dynamics of different diseases [51,52,53,70,71], as well as the effects on health after exposure to pesticides [25], allowing us to study through mathematical models the impact of prevention and mitigation campaigns considering individuals’ awareness.

## 5. Conclusions

Malaria disease represents a major health problem that has led to the implementation of several control measures, of which insecticide-treated nets (ITNs) have proven to be successful in reducing malaria in the last decades. However, the toxicity of the insecticides in the nets and misusing ITNs, for instance, for fishing, have been of concern for their potential capacity to harm human and ecosystem health. We present a compartmental model of impulsive differential equations to study the joint dynamics of malaria disease, acute and chronic intoxications in humans due to pulse ITN exposure, and collateral effects on ecosystems due to pulse ITN fishing, in combination with human risk perception to disease, intoxication, and ecosystem damage. Our results show that the point prevalence of malaria decreases significantly when increasing ITN coverage. However, this implies an increase in the cumulative number of intoxications, which is significant if the risk perception towards poisoning is low. Specifically, regarding risk perception levels, we can observe that the risk perception towards pesticides increases with increasing net coverage, contrary to the risk perception towards malaria disease, which decreases with increasing net coverage, and the risk perception towards collateral damage, which is much less impacted by coverage. Awareness campaigns on malaria and intoxications have an important impact on the reduction of malaria disease point prevalence and the cumulative number of intoxications. However, the same level of impact cannot be observed through awareness campaigns concerning the damage to the ecosystem due to the misuse of ITNs for fishing, i.e., they may not significantly reduce collateral damage. Therefore, we highlight the effect of restrictive measures taken to monitor the misuse of ITNs, and observe a greater reduction in collateral damage to the ecosystem when measures such as introducing closed fishing periods are implemented.

## Figures and Tables

**Table 1:** Description of variables for the model given in system (5).

Variable	Definition	Units *
**Humans**		
$S_{h},S_{h}^{p},S_{h}^{w}$	Malaria-susceptible individuals w/o, w/ acute, w/ chronic intoxication	H
$E_{h},E_{h}^{p},E_{h}^{w}$	Malaria-exposed individuals w/o, w/ acute, w/ chronic intoxication	H
$I_{h},I_{h}^{p},I_{h}^{w}$	Malaria-infected individuals w/o, w/ acute, w/ chronic intoxication	H
$R_{h},R_{h}^{p},R_{h}^{w}$	Malaria-recovered individuals w/o, w/ acute, w/ chronic intoxication	H
$K_{p}$	Risk perception to ITNs toxicity	R
$K_{d}$	Risk perception to malaria disease	R
$K_{c}$	Risk perception to collateral ecosystem damage from misuse of ITNs	R
**Vector**		
$S_{v},S_{h}^{r}$	Malaria susceptible mosquito w/o, w/ insecticide resistance	M
$I_{v},I_{h}^{r}$	Malaria infected mosquito w/o, w/ insecticide resistance	M
**Collateral effect—aquatic ecosystem**		
$S_{c}$	Ecosystem susceptible to damage	C
$D_{c}$	Damaged ecosystem	C

* H = humans, M = mosquitoes, C = ecosystem species, R = risk perception, w/ = with, w/o = without.

**Table 2:** Description of parameters and parameter value ranges for the model given in system (5) and used in the simulations.

Parameter	Definition	Baseline	Units *	Reference
**Humans**				
$\beta_{h,a}$	Malaria transmission rate to human	[0.015, 0.22]	D${}^{-1}$	[54,55,56,57,58]
$1/\delta_{h}$	Average incubation time	[12, 30]	D	[55,58]
$1/\gamma_{h}$	Mean infectious period	[180, 720]	D	[54,55,56,57,58]
$\omega_{h}$	Loss of immunity rate	$\left[5.5,1100\right]*10^{-5}$	D${}^{-1}$	[54]
μ	Intoxication proportion	[0, 0.3]	U	[26,30]
1/f	Mean detoxification period	[1/6, 2]	D	[59,60]
*g*	Health effects after prolonged exposure	0.00289	D${}^{-1}$	[25,26]
ϕ	Recovery rate from chronic toxicity	[0, 0.5]	D${}^{-1}$	Author chosen
$\Lambda_{h}$	Recruitment rate	$N_{h}*d_{h}$	HD${}^{-1}$	Author chosen
$d_{h}$	Natural mortality rate	$\left[3.3,5.5\right]*10^{-5}$	D${}^{-1}$	[61,62]
${\hat{d}}_{h}$	Disease-induced mortality rate	$\left[0,4.1\right]*10^{-4}$	D${}^{-1}$	[54]
$\lambda_{1}^{d,p,c}$	Rate of resistance to change risk perception	[0, 1]	D${}^{-1}$	[25,51,52,53]
$\lambda_{2}^{d,p},\lambda_{2}^{c}$	Per capita reaction to change risk perception	[0, 1]	RD${}^{-1}$, R	[25,51,52,53]
η	Mosquito net coverage	[0, 1]	U	[7]
**Vector**				
$\beta_{v,a}$	Malaria transmission rate to mosquito	[0.015, 0.24]	D${}^{-1}$	[54,55,56,57,58]
$\sigma_{}$	Proportion of mosquitoes becoming resistant	[0, 1]	U	[1,7,63]
$\Lambda_{v}$	Recruitment rate	$N_{v}*d_{v}$	MD${}^{-1}$	Author chosen
$d_{v}$	Natural mortality rate	[1/30, 1]	D${}^{-1}$	[57,64]
θ	Increase in the mortality rate	[4.4, 48.9]%	U	[65]
**Collateral effect—aquatic ecosystem**				
Ω	Percentage of species susceptible to ITN fishing	0.75	U	[66]
$\Lambda_{c}$	Recruitment rate	$d_{c}*N_{c}$	CD${}^{-1}$	Author chosen
$d_{c}$	Natural mortality rate	$\left[8.4,11.5\right]*10^{-4}$	D${}^{-1}$	[67,68]
${\hat{d}}_{c}$	Damage-induced mortality rate	$d_{c}+\left[14.7,21\right]*10^{-4}$	D${}^{-1}$	[67,68]
ψ	Loss of damage rate	$4.2*10^{-3}$	D${}^{-1}$	[67,68]

* D = days, U = unitless, H = humans, M = mosquitoes, C = ecosystem species, R = risk perception.

## Data Availability

Not applicable.

## Abbreviations

The following abbreviations are used in this manuscript:

ITN	Insecticide-treated net
WHO	World Health Organization

## References

[1] World Health Organization (WHO) Malaria. https://www.who.int/news-room/fact-sheets/detail/malaria.

[2] Centers for Disease Control and Prevention (CDC) About Malaria. https://www.cdc.gov/malaria/about/.

[3] Almaw A., Yimer M., Alemu M., Tegegne B. (2022). Prevalence of malaria and associated factors among symptomatic pregnant women attending antenatal care at three health centers in north-west Ethiopia. PLoS ONE.

[4] Uneke C.J., Ogbu O., Nwojiji V. (2006). Potential risk of induced malaria by blood transfusion in South-eastern Nigeria. McGill J. Med. MJM.

[5] Cabrera M., Taylor G., Saldaña-Núñez V., Córdova-Lepe F., Escalera-Antezana J.P., Alvarado-Arnez L.E., Rodríguez-Morales A.J. (2019). Risk of Dengue Incidence in Children and Adolescents in Zulia, Venezuela, using a Negative Binomial Generalized Linear Mixed Model. Rev. Panam. Enferm. Infecc..

[6] Guyatt H.L., Snow R.W. (2004). Impact of malaria during pregnancy on low birth weight in sub-Saharan Africa. Clin. Microbiol. Rev..

[7] Roll Back Malaria. https://endmalaria.org/about-malaria/key-facts.

[8] World Health Organization (WHO) World Malaria Report 2021. https://www.who.int/publications/i/item/9789240040496.

[9] Pryce J., Medley N., Choi L. (2022). Indoor residual spraying for preventing malaria in communities using insecticide-treated nets. Cochrane Database Syst. Rev..

[10] Sherrard-Smith E., Winskill P., Hamlet A., Ngufor C., N’Guessan R., Guelbeogo M.W., Sanou A., Nash R.K., Hill A., Russell E.L. (2022). Optimising the deployment of vector control tools against malaria: A data-informed modelling study. Lancet Planet. Health.

[11] Pryce J., Richardson M., Lengeler C. (2018). Insecticide-treated nets for preventing malaria. Cochrane Database Syst. Rev..

[12] Wetzler E.A., Park C., Arroz J.A., Chande M., Mussambala F., Candrinho B. (2022). Impact of mass distribution of insecticide-treated nets in Mozambique, 2012 to 2025: Estimates of child lives saved using the Lives Saved Tool. PLoS Glob. Public Health.

[13] Millat-Martínez P., Gabong R., Balanza N., Luana S., Sanz S., Raulo S., Elizah A., Wali C., Paivu B., Dalmas J. (2021). Coverage, determinants of use and repurposing of long-lasting insecticidal nets two years after a mass distribution in Lihir Islands, Papua New Guinea: A cross-sectional study. Malar. J..

[14] Larsen D.A., Makaure J., Ryan S.J., Stewart D., Traub A., Welsh R., Love D.H., Bisesi J.H. (2021). Implications of insecticide-treated mosquito net fishing in lower income countries. Environ. Health Perspect..

[15] McLean K.A., Byanaku A., Kubikonse A., Tshowe V., Katensi S., Lehman A.G. (2014). Fishing with bed nets on Lake Tanganyika: A randomized survey. Malar. J..

[16] Short R., Gurung R., Rowcliffe M., Hill N., Milner-Gulland E. (2018). The use of mosquito nets in fisheries: A global perspective. PLoS ONE.

[17] Berthe S., Harvey S.A., Lynch M., Koenker H., Jumbe V., Kaunda-Khangamwa B., Mathanga D.P. (2019). Poverty and food security: Drivers of insecticide-treated mosquito net misuse in Malawi. Malar. J..

[18] Jones B.L., Unsworth R.K. (2020). The perverse fisheries consequences of mosquito net malaria prophylaxis in East Africa. Ambio.

[19] World Health Organization (WHO) (2022). Guidelines for Malaria. https://apps.who.int/iris/bitstream/handle/10665/354781/WHO-UCN-GMP-2022.01-Rev.2-eng.pdf.

[20] (2018). RBM Partnership to End Malaria, Consensus Statement on Repurposing ITNs: Applications for BCC Messaging and Actions at the Country Level. https://pdf.usaid.gov/pdf_docs/PA00TW8X.pdf.

[21] USAID Programmatic Environmental Assessment: Integrated Vector Management Programs for Malaria Vector Control (version 2017). https://d1u4sg1s9ptc4z.cloudfront.net/uploads/2021/03/integrated-vector-management-programs-for-malaria-vector-control-programmatic-environmental-assessment-2017-2.pdf.

[22] Hu R., Huang X., Huang J., Li Y., Zhang C., Yin Y., Chen Z., Jin Y., Cai J., Cui F. (2015). Long- and Short-Term Health Effects of Pesticide Exposure: A Cohort Study from China. PLoS ONE.

[23] Kim J.Y., Park S., Kim S.K., Kim C.S., Kim T.H., Min S.H., Oh S.S., Koh S.B. (2019). Correction: Pesticide exposure and cognitive decline in a rural South Korean population. PLoS ONE.

[24] Liu J., Schelar E. (2012). Pesticide Exposure and Child Neurodevelopment: Summary and Implications. Workplace Health Saf..

[25] Muñoz-Quezada M.T., Lucero B.A., Gutiérrez-Jara J.P., Buralli R.J., Zúñiga-Venegas L., Muñoz M.P., Ponce K.V., Iglesias V. (2020). Longitudinal exposure to pyrethroids (3-PBA and trans-DCCA) and 2,4-D herbicide in rural schoolchildren of Maule region, Chile. Sci. Total Environ..

[26] Zúñiga-Venegas L.A., Hyland C., Muñoz-Quezada M.T., Quirós-Alcalá L., Butinof M., Buralli R., Cardenas A., Fernandez R.A., Foerster C., Gouveia N. (2022). Health Effects of Pesticide Exposure in Latin American and the Caribbean Populations: A Scoping Review. Environ. Health Perspect..

[27] Anyanwu E.C., Ehiri J.E., Kanu I., Merrick J. (2006). Health effects of long-term exposure to insecticide-treated mosquito nets in the control of malaria in endemic regions, revised. Sci. World J..

[28] Kolaczinski J., Curtis C. (2004). Chronic illness as a result of low-level exposure to synthetic pyrethroid insecticides: A review of the debate. Food Chem. Toxicol..

[29] Bomann W. (1995). How safe are pyrethroid-treated mosquito nets? An evaluation based on the example of Solfac EW 050. Public Health.

[30] Lu G., Traoré C., Meissner P., Kouyaté B., Kynast-Wolf G., Beiersmann C., Coulibaly B., Becher H., Müller O. (2015). Safety of insecticide-treated mosquito nets for infants and their mothers: Randomized controlled community trial in Burkina Faso. Malar. J..

[31] Brauer F., Castillo-Chavez C., Mubayi A., Towers S. (2016). Some models for epidemics of vector-transmitted diseases. Infect. Dis. Model..

[32] Simoy M.I., Aparicio J.P. (2020). Ross-Macdonald models: Which one should we use?. Acta Trop..

[33] Gutiérrez-Jara J.P., Salazar-Viedma M., González C.R., Cancino-Faure B. (2022). The emergence of Dirofilaria repens in a non-endemic area influenced by climate change: Dynamics of transmission using a mathematical model. Acta Trop..

[34] Ross R. (1915). Some a priori pathometric equations. Br. Med. J..

[35] Ngonghala C.N. (2022). Assessing the impact of insecticide-treated nets in the face of insecticide resistance on malaria control. J. Theor. Biol..

[36] Ndamuzi E., Gahungu P. (2021). Mathematical Modeling of Malaria Transmission Dynamics: Case of Burundi. J. Appl. Math. Phys..

[37] Witbooi P., Abiodun G., Nsuami M. (2021). A model of malaria population dynamics with migrants. Math. Biosci. Eng..

[38] Vogt-Geisse K., Ngonghala C.N., Feng Z. (2020). The impact of vaccination on malaria prevalence: A vaccine-age-structured modeling approach. J. Biol. Syst..

[39] Vogt-Geisse K., Lorenzo C., Feng Z. (2013). Impact of age-dependent relapse and immunity on malaria dynamics. J. Biol. Syst..

[40] Mandal S., Sarkar R.R., Sinha S. (2011). Mathematical models of malaria-a review. Malar. J..

[41] Lindsay S.W., Thomas M.B., Kleinschmidt I. (2021). Threats to the effectiveness of insecticide-treated bednets for malaria control: Thinking beyond insecticide resistance. Lancet Glob. Health.

[42] White L.J., Maude R.J., Pongtavornpinyo W., Saralamba S., Aguas R., Van Effelterre T., Day N.P., White N.J. (2009). The role of simple mathematical models in malaria elimination strategy design. Malar. J..

[43] Chitnis N., Schapira A., Smith T., Steketee R. (2010). Comparing the effectiveness of malaria vector-control interventions through a mathematical model. Am. J. Trop. Med. Hyg..

[44] Agusto F.B., Del Valle S.Y., Blayneh K.W., Ngonghala C.N., Goncalves M.J., Li N., Zhao R., Gong H. (2013). The impact of bed-net use on malaria prevalence. J. Theor. Biol..

[45] Chinebu T.I., Ezennorom E.O., Okwor J.U. (2018). Simulation of a Mathematical Model of Malaria Transmission Dynamics in the Presence of Mosquito Net, Fumigation And Treatment. Int. J. Trend Sci. Res. Dev..

[46] Aldila D. (2022). Dynamical Analysis on a Malaria Model with Relapse Preventive Treatment and Saturated Fumigation. Comput. Math. Methods Med..

[47] Córdova-Lepe F., Pinto M., González-Olivares E. (2012). A new class of differential equations with impulses at instants dependent on preceding pulses. Applications to management of renewable resources. Nonlinear Anal. Real World Appl..

[48] Liu H., Yu J., Zhu G. (2006). Analysis of a vector-host malaria model with impulsive effect and infection-age. Adv. Complex Syst..

[49] Huang M., Song X., Li J. (2017). Modelling and analysis of impulsive releases of sterile mosquitoes. J. Biol. Dyn..

[50] Gutiérrez-Jara J.P., Córdova-Lepe F., Muñoz-Quezada M.T., Chowell G. (2020). Pesticide application, educational treatment and infectious respiratory diseases: A mechanistic model with two impulsive controls. PLoS ONE.

[51] Cabrera M., Cordova-Lepe F., Gutierrez-Jara J.P., Vogt Geisse K. (2021). An SIR type epidemiological model that integrates social distancing as a dynamic law based on point prevalence and socio-behavioral factors. Sci. Rep..

[52] Gutiérrez-Jara J.P., Vogt Geisse K., Cabrera M., Cordova-Lepe F., Muñoz-Quezada M.T. (2022). Effects of human mobility and behavior on disease transmission in a COVID-19 mathematical model. Sci. Rep..

[53] Gutiérrez-Jara J.P., Saracini C. (2022). Risk Perception Influence on Vaccination Program on COVID-19 in Chile: A Mathematical Model. Int. J. Environ. Res. Public Health.

[54] Chitnis N., Hyman M.J., Cushing J.M. (2008). Determining Important Parameters in the Spread of Malaria Through the Sensitivity Analysis of a Mathematical Model. Bull. Math. Biol..

[55] Nah K., Kim Y., Lee J.M. (2010). The dilution effect of the domestic animal population on the transmission of P. vivax malaria. J. Theor. Biol..

[56] de Alencar F.E., Malafronte R.D.S., Cerutti Junior C., Natal Fernandes L., Buery J.C., Fux B., Rezende H.R., Duarte A.M.R.D.C., Medeiros-Sousa A.R., Miranda A.E. (2021). Assessment of asymptomatic Plasmodium spp. infection by detection of parasite DNA in residents of an extra-Amazonian. Malar. J..

[57] Medeiros-Sousa A.R., Laporta G.Z., Coutinho R.M., Mucci L.F., Marrelli M.T. (2021). A mathematical model for zoonotic transmission of malaria in the Atlantic Forest: Exploring the effects of variations in vector abundance and acrodendrophily. PLoS Negl. Trop. Dis..

[58] Kim S., Byun J.H., Park A., Jung I.H. (2020). A mathematical model for assessing the effectiveness of controlling relapse in Plasmodium vivax malaria endemic in the Republic of Korea. PLoS ONE.

[59] Agency for Toxic Substances and Disease Registry (ATSDR) Toxical Profile for Pyrethrins and Pyrethroids. https://www.atsdr.cdc.gov/toxprofiledocs/index.html?id=787&tid=153.

[60] Rodríguez J.L., Ares I., Martínez M., Martínez-Larrañaga M.R., Anadón A., Martínez M.A. (2018). Bioavailability and nervous tissue distribution of pyrethroid insecticide cyfluthrin in rats. Food Chem. Toxicol..

[61] World Bank Data, Life Expectancy at Birth, Total (Years)—Sub-Saharan Africa. https://data.worldbank.org/indicator/SP.DYN.LE00.IN?locations=ZG.

[62] The World Factbook, Central Intelligence Agency Life Expectancy at Birth. https://www.cia.gov/the-world-factbook/field/life-expectancy-at-birth/country-comparison.

[63] Insecticide-Treated Mosquito Net Interventions: A Manual for National Control Programme Managers. Edited by Roll Back Malaria. https://apps.who.int/iris/handle/10665/42685.

[64] Laporta G.Z., Prado P.I.K.L.d., Kraenkel R.A., Coutinho R.M., Sallum M.A.M. (2013). Biodiversity Can Help Prevent Malaria Outbreaks in Tropical Forests. PLoS Negl. Trop. Dis..

[65] Kouassi B.L., Edi C., Tia E., Konan L.Y., Akré M.A., Koffi A.A., Ouattara A.F., Tanoh A.M., Zinzindohoue P., Kouadio B. (2020). Susceptibility of Anopheles gambiae from Côte d’Ivoire to insecticides used on insecticide-treated nets: Evaluating the additional entomological impact of piperonyl butoxide and chlorfenapyr. Malar. J..

[66] Mwandya A.W., Gullström M., Öhman M.C., Andersson M.H., Mgaya Y.D. (2009). Fish assemblages in Tanzanian mangrove creek systems influenced by solar salt farm constructions. Estuar. Coast. Shelf Sci..

[67] El Ganainy A., Khouraiba H., Aly M., Osman M.A. (2020). Fishery assessment of the common silver biddy gerres oyena from the gulf of Suez, Red Sea, Egypt. Egypt. J. Aquat. Biol. Fish..

[68] Yongo E., Agembe S., Outa N., Owili M. (2018). Growth, mortality and recruitment of Nile perch (Lates niloticus) in Lake Victoria, Kenya. Lakes Reserv. Res. Manag..

[69] Owusu V., Andriesse E. (2020). From open access regime to closed fishing season: Lessons from small-scale coastal fisheries in the Western Region of Ghana. Mar. Policy.

[70] Cabrera M., Taylor G. (2019). Modelling spatio-temporal data of dengue fever using generalized additive mixed models. Spat. Spatio-Temporal Epidemiol..

[71] Córdova-Lepe F., Vogt-Geisse K. (2022). Adding a reaction-restoration type transmission rate dynamic-law to the basic SEIR COVID-19 model. PLoS ONE.

